# Supplementary material for: The fast-evolving FIKK kinase family of Plasmodium falciparum can be inhibited by a single compound
Source: Nat Microbiol. 2025 May 19;10(6):1463–83. doi: 10.1038/s41564-025-02017-4 (PMC12137140; doi:10.1038/s41564-025-02017-4)

nature microbiology

Supplementary information https://doi.org/10.1038/s41564-025-02017-4

The fast-evolving FIKK kinase family of *Plasmodium falciparum* can be inhibited by a single compound

In the format provided by the authors and unedited

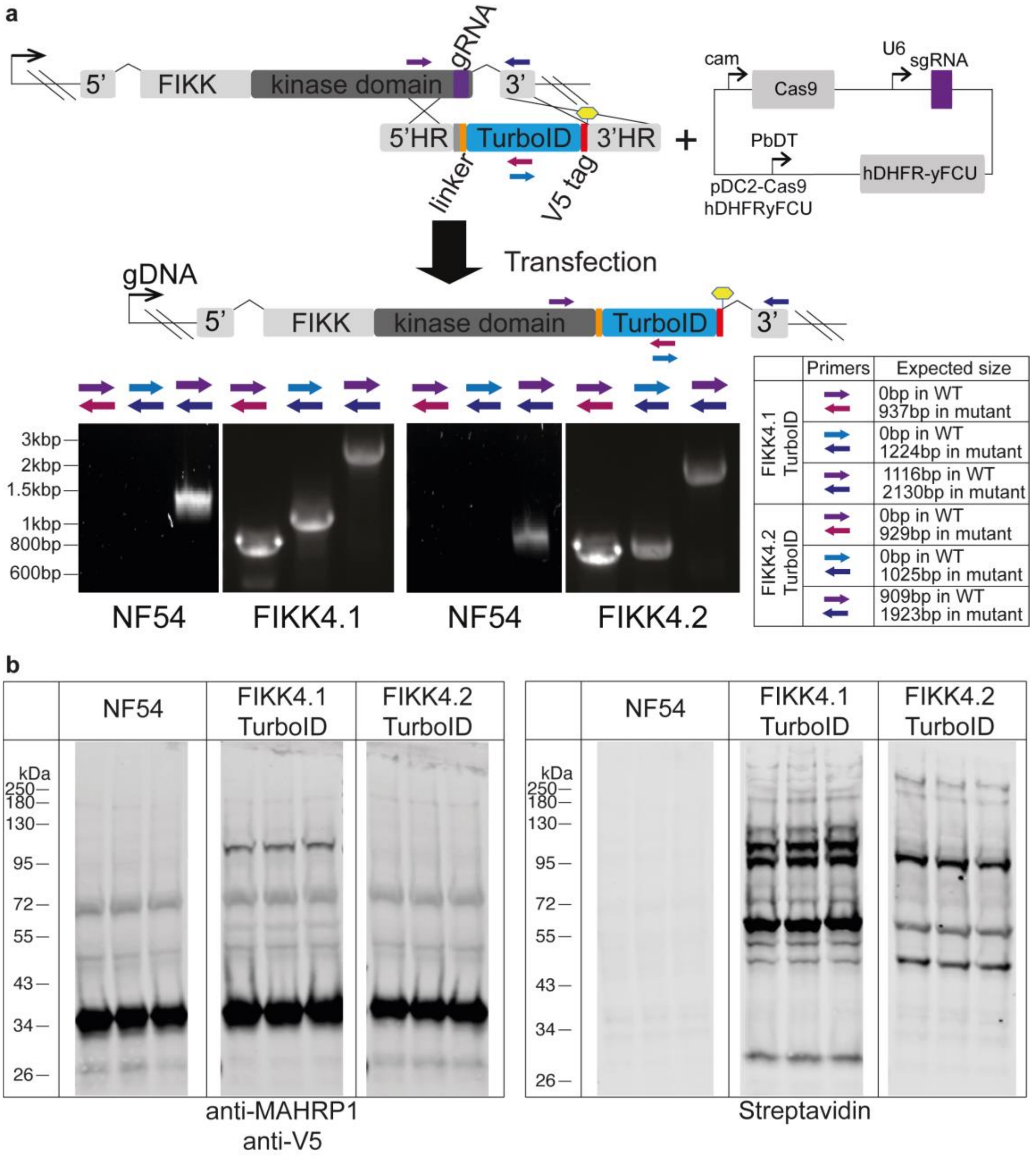

Supplementary Fig. 1. CRISPR/Cas9 strategy to generate FIKK::TurboID fusion proteins and validation.

a, Diagram illustrating the CRISPR/Cas9 strategy[1] used to insert a TurboID_V5 cassette at the C-terminal end of the *fikk* genes. Homology regions used to edit the genome are denoted by 5' and 3'HR and the Cas9 guide is denoted as a purple cassette. Yellow hexagons denote a stop codon. Primers used to investigate integration into the correct endogenous loci along with the presence of WT parasites into the mutant population are shown. Expected band size for PCR reactions are indicated. Material used to generate and validate FIKK::TurboID lines can be found in Supplementary Table 13. **b,** Western blots of cloned parental NF54, FIKK4.1::TurboID (111kDa) and FIKK4.2::TurboID (180kDa) fusion lines cultured in biotin-containing medium for the duration of the parasite asexual lifecycle (48h) probed with anti-V5 and streptavidin-fluorophore. Anti-MAHRP1 (29kDa) antibody is used as a loading control. This experiment was performed three times with similar results.

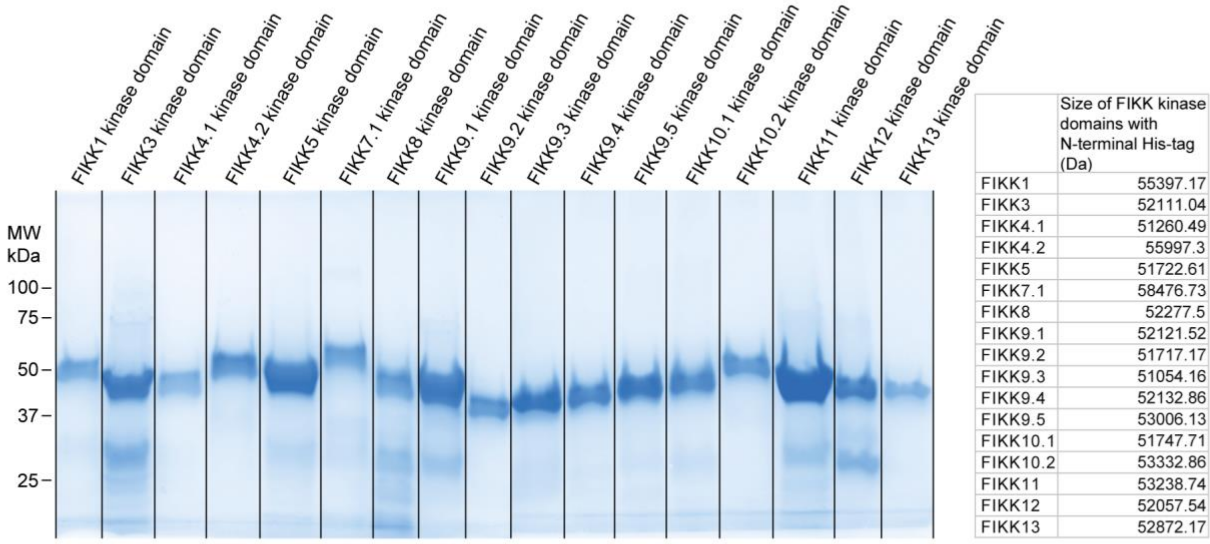

	Size of FIKK kinase domains with N-terminal His-tag (Da)
FIKK1	55397.17
FIKK3	52111.04
FIKK4.1	51260.49
FIKK4.2	55997.3
FIKK5	51722.61
FIKK7.1	58476.73
FIKK8	52277.5
FIKK9.1	52121.52
FIKK9.2	51717.17
FIKK9.3	51054.16
FIKK9.4	52132.86
FIKK9.5	53006.13
FIKK10.1	51747.71
FIKK10.2	53332.86
FIKK11	53238.74
FIKK12	52057.54
FIKK13	52872.17

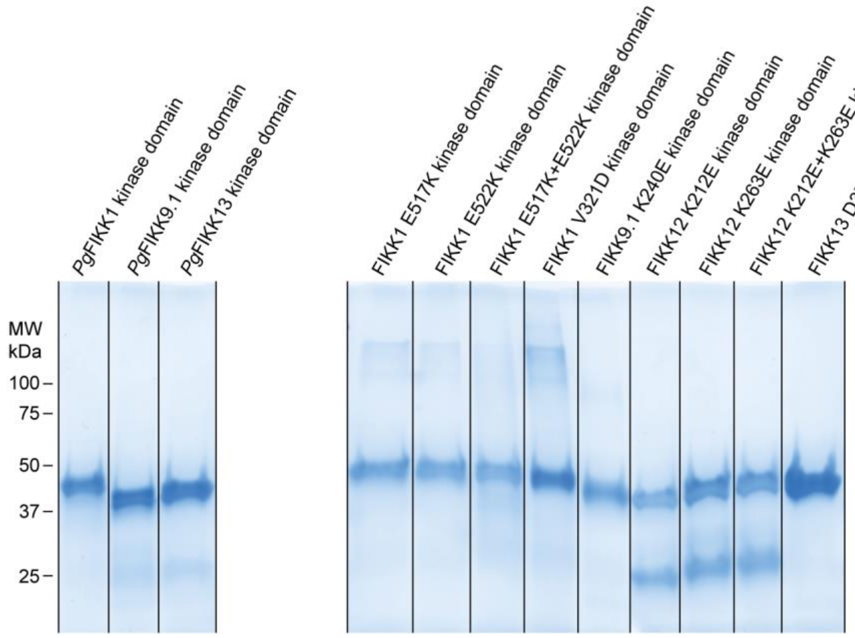

	Size of FIKK kinase domains with N-terminal His-tag (Da)
*Pg*FIKK1	54896.42
*Pg*FIKK9.1	52300.29
*Pg*FIKK13	53205.79
FIKK1 E517K	55396.34
FIKK1 E522K	55396.34
FIKK1 E517K+E522K	55395.40
FIKK1 V321D	55413.24
FIKK9.1 K240E	52122.62
FIKK12 K212E	52058.98
FIKK12 K263E	52058.98
FIKK12 K212E+K263E	52059.92
FIKK13 D379N	52871.19

Supplementary Fig. 2. Coomassie-stained gel of purified recombinant FIKK kinase domains.
Protein ladder is depicted on the left-hand side of the gel in kilodaltons (kDa). Predicted sizes of the purified recombinant kinase domains with N-terminal His-tag are indicated in Dalton in the tables.

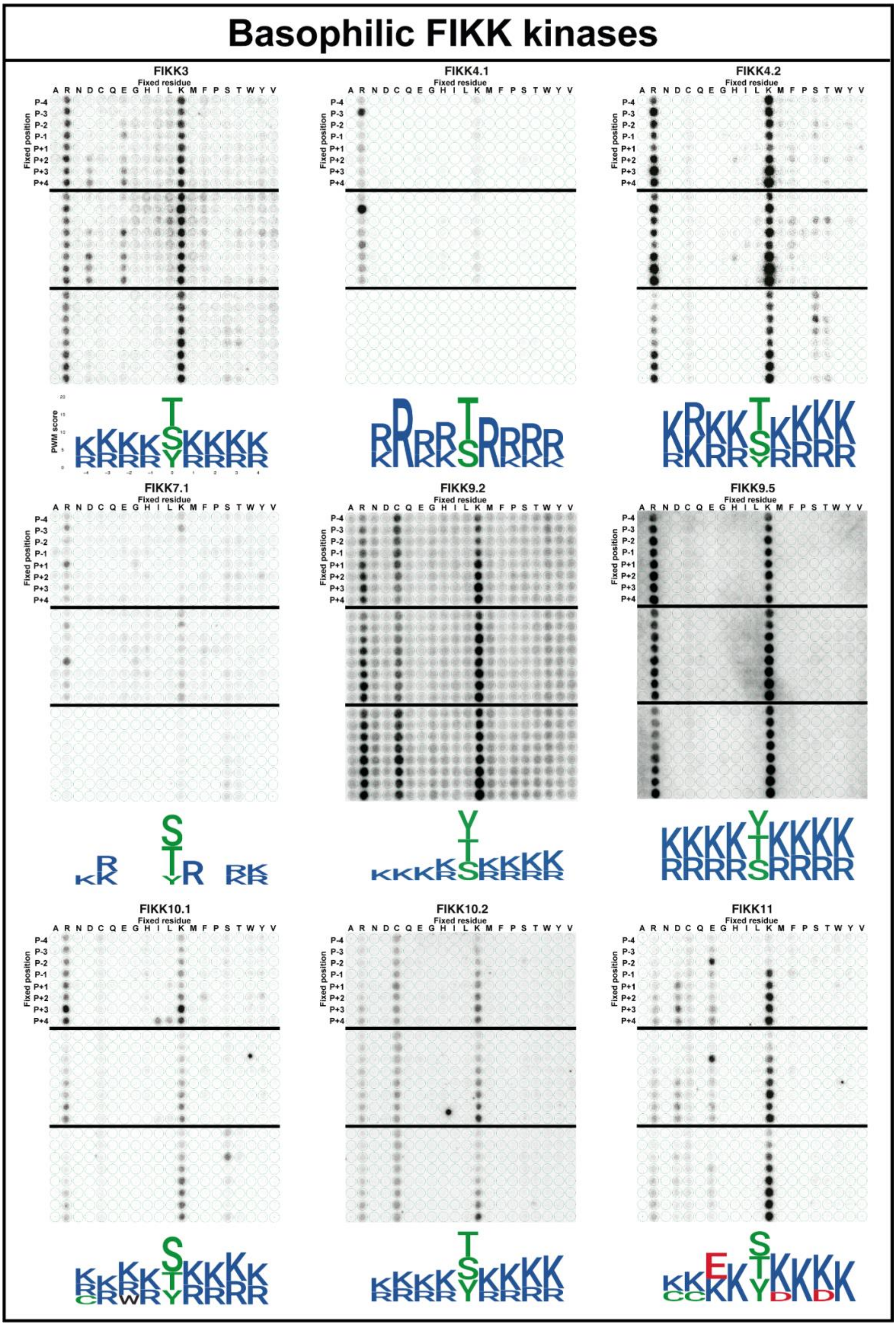

Supplementary Fig. 3. Basophilic FIKK kinases preferred phosphorylation motifs.

See Extended Data Fig. 3 caption.

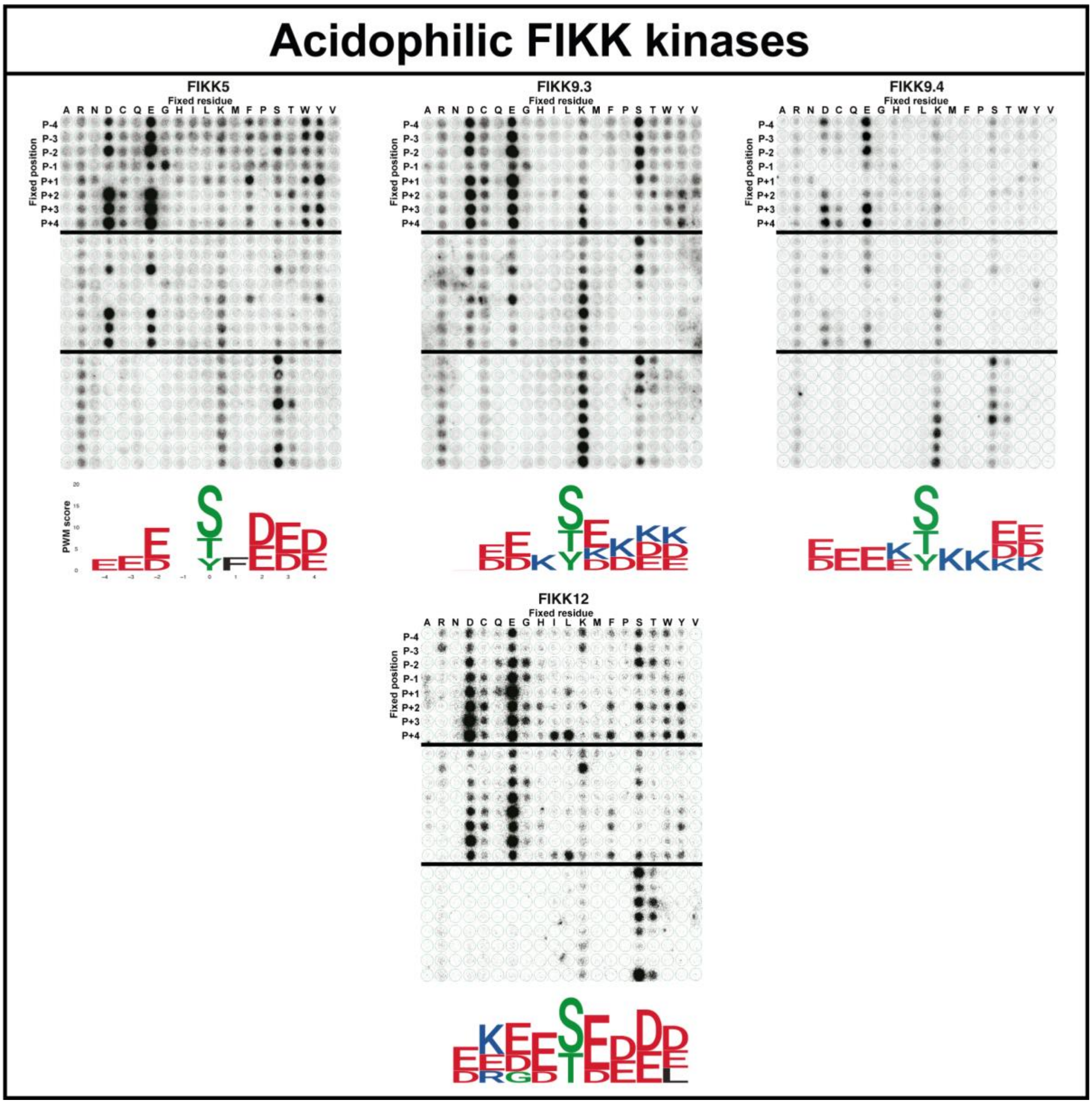

Supplementary Fig. 4. Acidophilic FIKK kinases preferred phosphorylation motifs.

See Extended Data Fig. 3 caption.

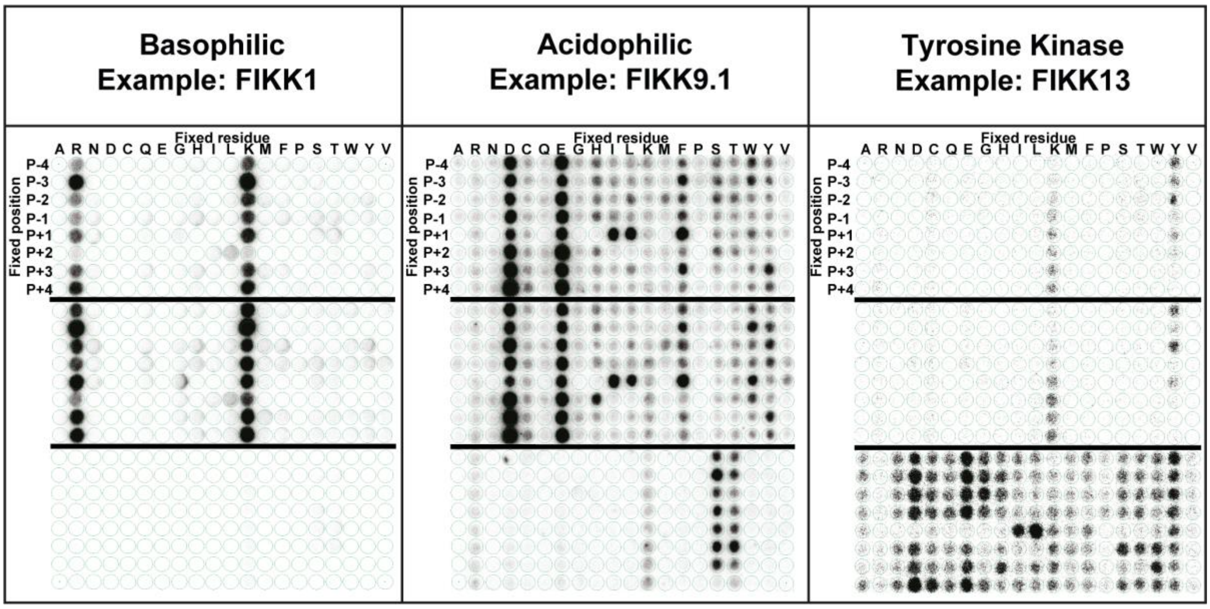

Supplementary Fig. 5. FIKK1, FIKK9.1 and FIKK13 OPAL membranes.
See Extended Data Fig. 3 caption.

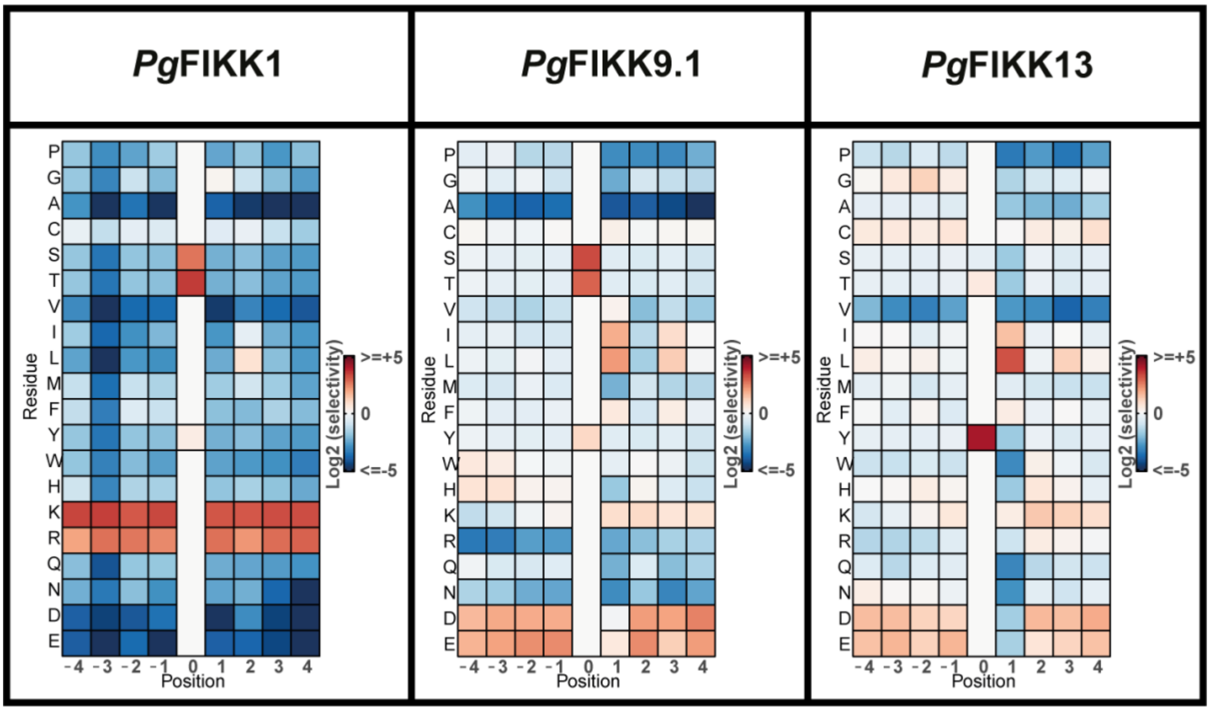

Supplementary Fig. 6. Heatmap representation of OPAL arrays raw data for *P. gaboni* FIKK1, FIKK9.1 and FIKK13.

Heatmap representation of OPAL array raw data for *P. gaboni* FIKK1, FIKK9.1 and FIKK13. See Fig. 3 caption.

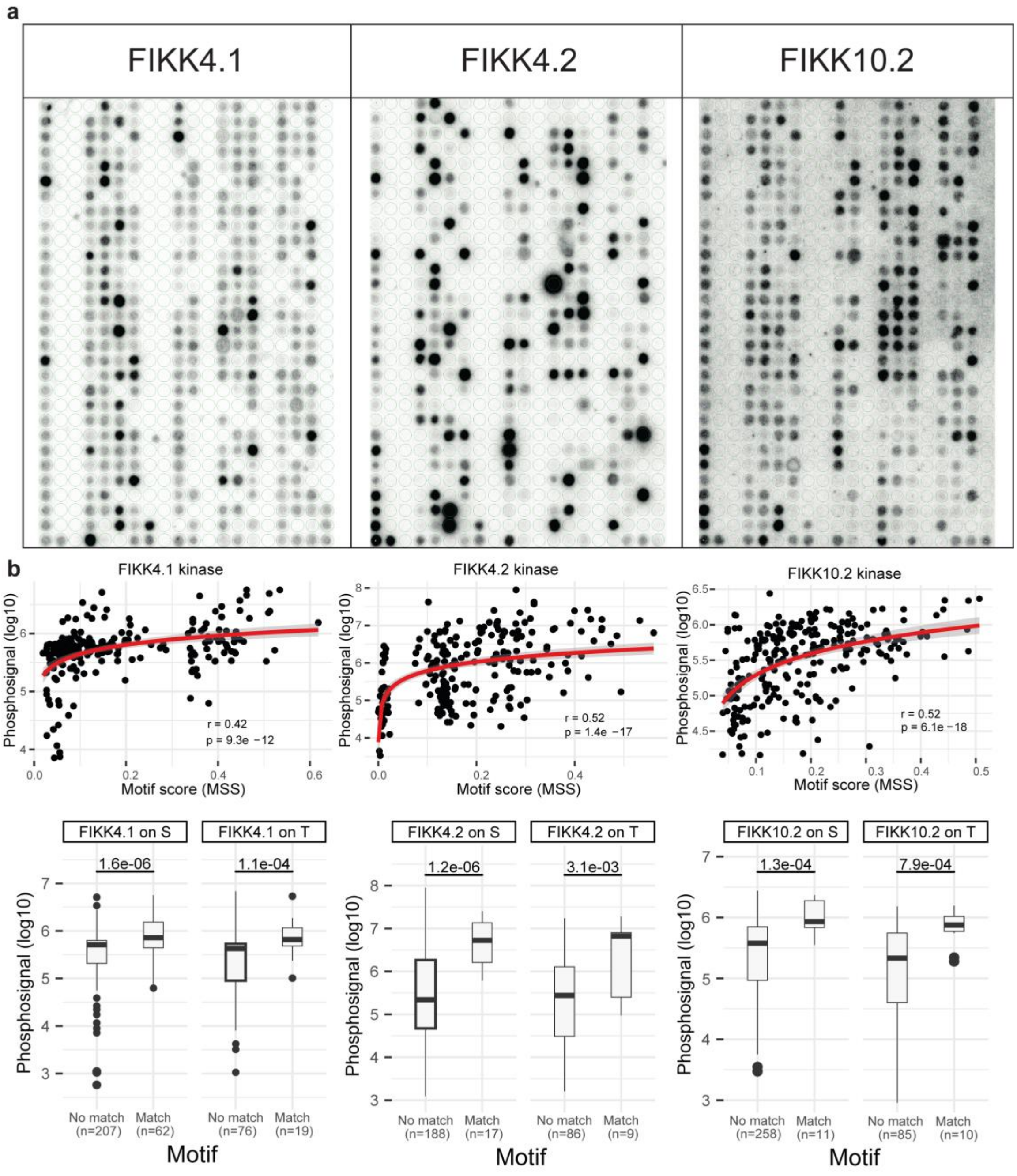

Supplementary Fig. 7. FIKK4.1, FIKK4.2 and FIKK10.2 activity on the phosphoproteome peptides libraries.

a, Phosphorimager scans of phosphorscreen exposed to phosphoproteome peptide membranes incubated with either recombinant FIKK4.1, FIKK4.2 or FIKK10.2 kinase domains and [γ-32P]-ATP. **b,** Top: correlation of FIKK kinase activity on the phosphoproteome peptide membrane (log10-scaled) against the corresponding FIKK motif score (matrix similarity score) for each peptide. For FIKK 4.1, FIKK 4.2, and FIKK 10.2 kinases. Pearson's correlation for the y = log(x) curve. Bottom: difference in FIKK phosphorylation signal (log10-scaled) between peptides without or with a match to the corresponding FIKK motif, for peptides with an S or T phosphoacceptor, for FIKK 4.1, FIKK 4.2, and FIKK 10.2 kinases. n represents the number of peptides. Centre line represents the median, box limits are upper and lower quartiles, whiskers represent 1.5x the interquartile range and each point indicates an outlier (Wilcoxon test, one-sided).

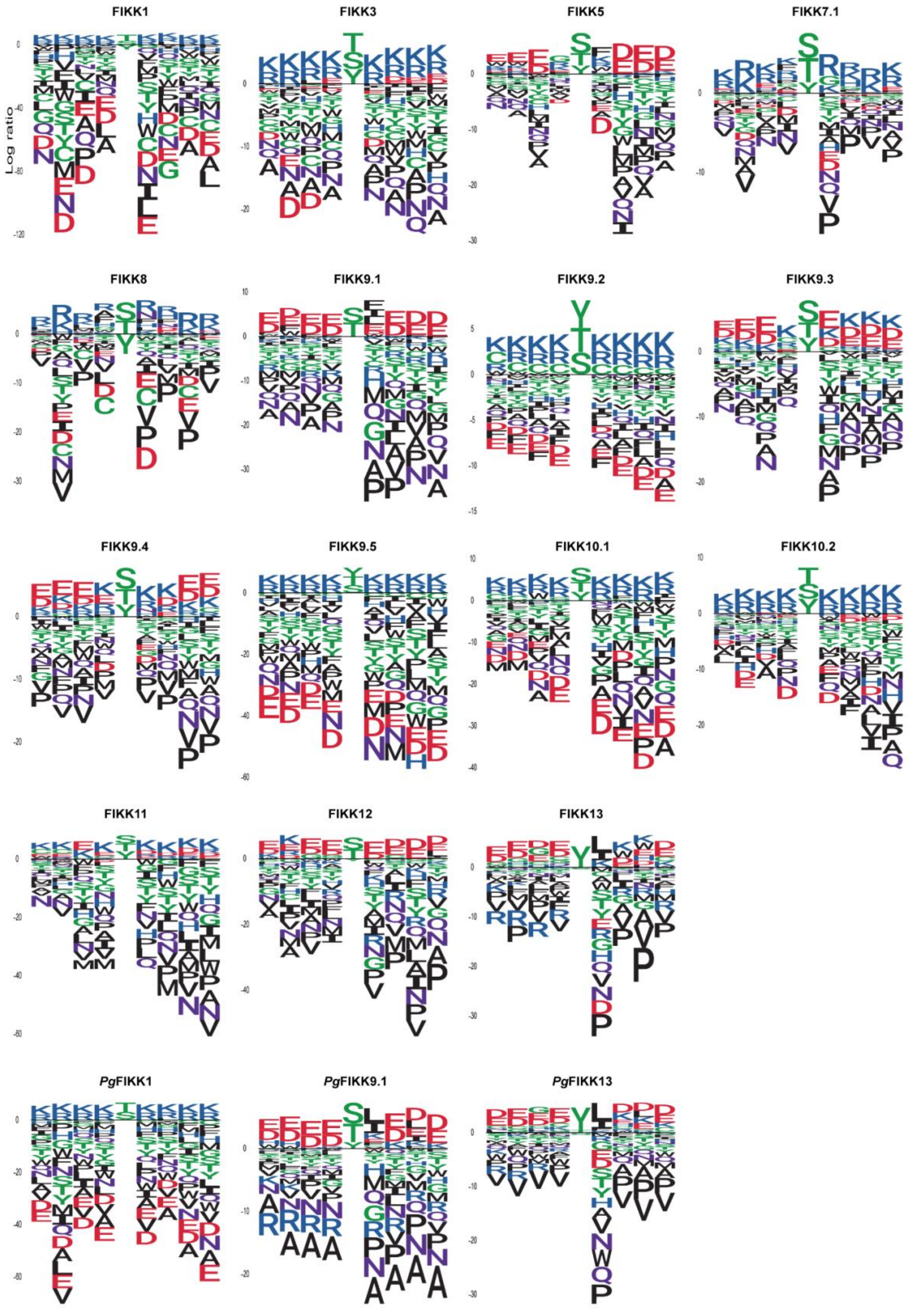

Supplementary Fig. 8. Log2 transformed PWM logos for all recombinant FIKK kinases tested.
See Fig. 4f caption.

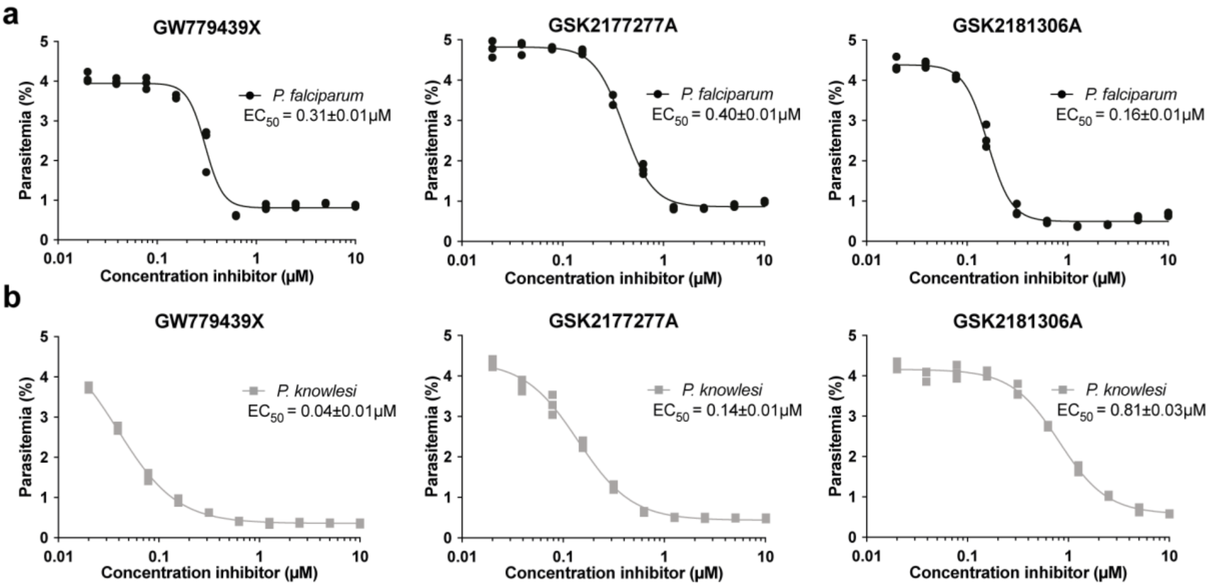

Supplementary Fig. 9. The three most potent *in vitro* FIKK inhibitors kill *Plasmodium* parasites in culture.

Half maximal effective concentration (EC_{50}) *in vitro* determination for GW779439X, GSK2177277A and GSK2181306A towards *P. falciparum* **a** and *P. knowlesi* **b** parasites. Parasitemia was assessed by flow cytometry using SYBR Green staining of the parasite nucleus after a 72 hours incubation period in the presence of different concentrations of compounds (parasitemia indicated in Supplementary Table 18). EC_{50}s were determined using a four-parameter dose-response model with the software PRISM. Data are shown as the mean±SEM for 3 biological replicates. Curves represent dose-response curves of FIKK inhibitors inhibition of *P. falciparum* (black) and *P. knowlesi* (grey).

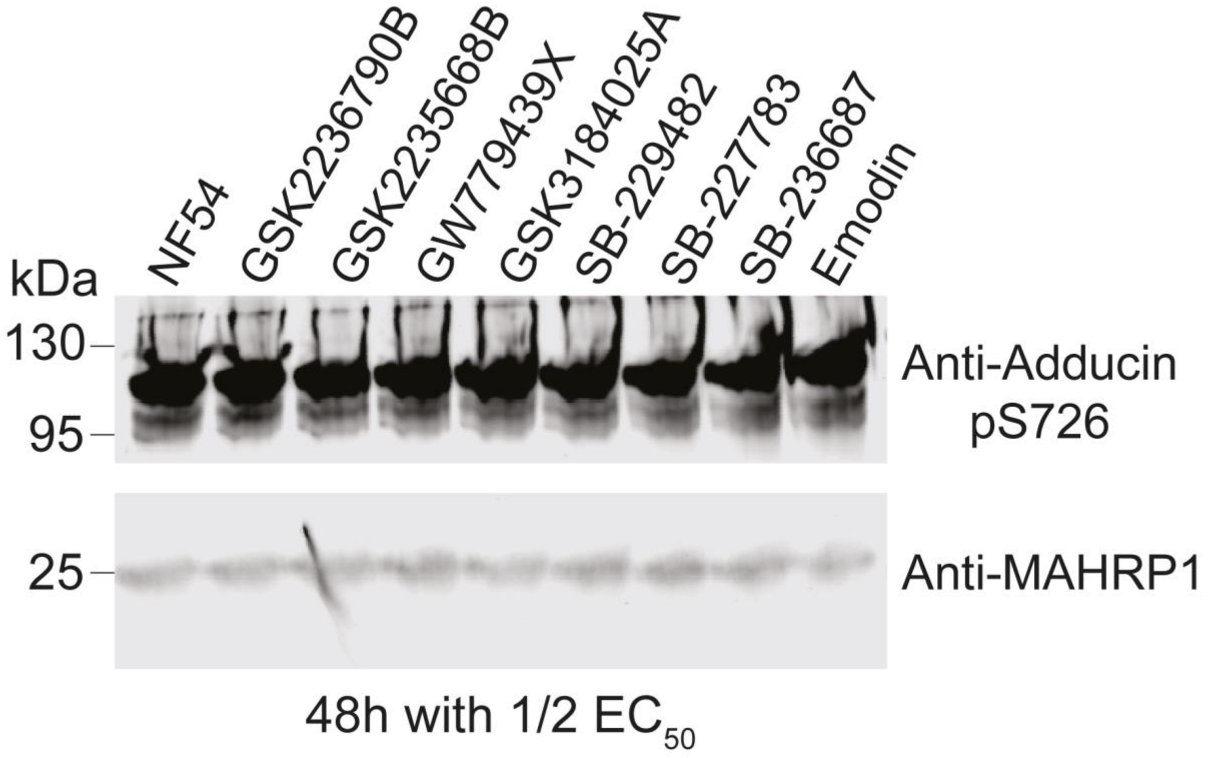

Supplementary Fig. 10. SAR-identified compounds and Emodin do not prevent Adducin S726 phosphorylation in normal RBCs.

Western blot showing adducin S726 phosphorylation in iRBCs treated with SAR-identified compounds and Emodin at 1⁄2 EC_{50} for 48 hours. The MAHRP1 antibody (bottom) demonstrates equal loading. This experiment was performed three times with similar results.

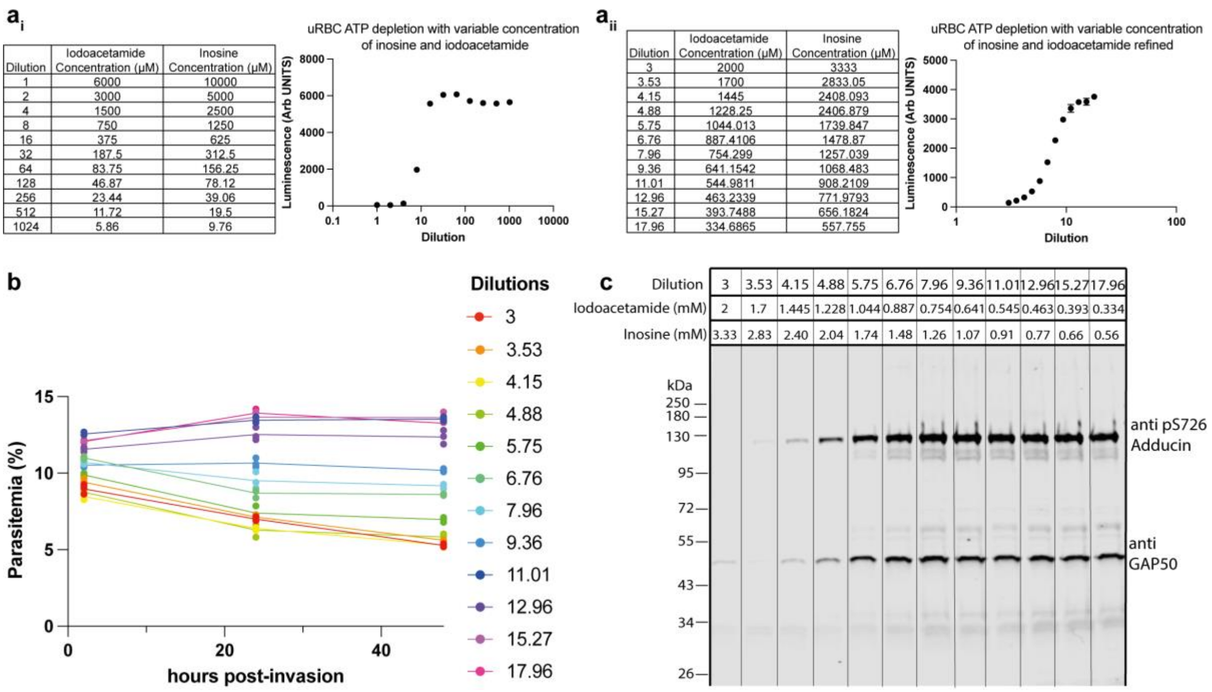

Supplementary Fig. 11. Optimisation of ATP-depletion conditions.

a, Measurement of intra-erythrocytic ATP concentrations in uRBC using the CellTiter-Glo→ luminescence assay (Promega). (i) Luminescence, relative to intra-erythrocytic ATP concentration, was measured in uRBC pre-treated with iodoacetamide and inosine concentrations ranging from 6000µM to 5.86µM and 10000µM to 9.76µM respectively. (ii) Luminescence measured in uRBC pre-treated with iodoacetamide and inosine concentrations ranging from 2000µM to 334.7µM and 3333µM to 557.8µM respectively corresponding to dilution 3 to 17.96 from (**a**i). n = 3 biological replicates for both (i) and (ii). **b,** Parasitemia assessed for NF54 iRBCs pre-treated with different concentrations of iodoacetamide and inosine corresponding to dilution 3 to 17.96 from (**a**ii). Parasitemia was assessed by flow cytometry using SYBR green staining and numerical values of percentages is provided in Supplementary Table 19. n=3 biological replicates. **c,** Western blot investigating adducin S726 phosphorylation in NF54 iRBCs pre-treated with different concentrations of iodoacetamide and inosine corresponding to dilution 3 to 17.96 from (**a**ii). Anti-GAP50 antibody is used here to investigate viability of the parasite. This experiment was performed three times with similar results.

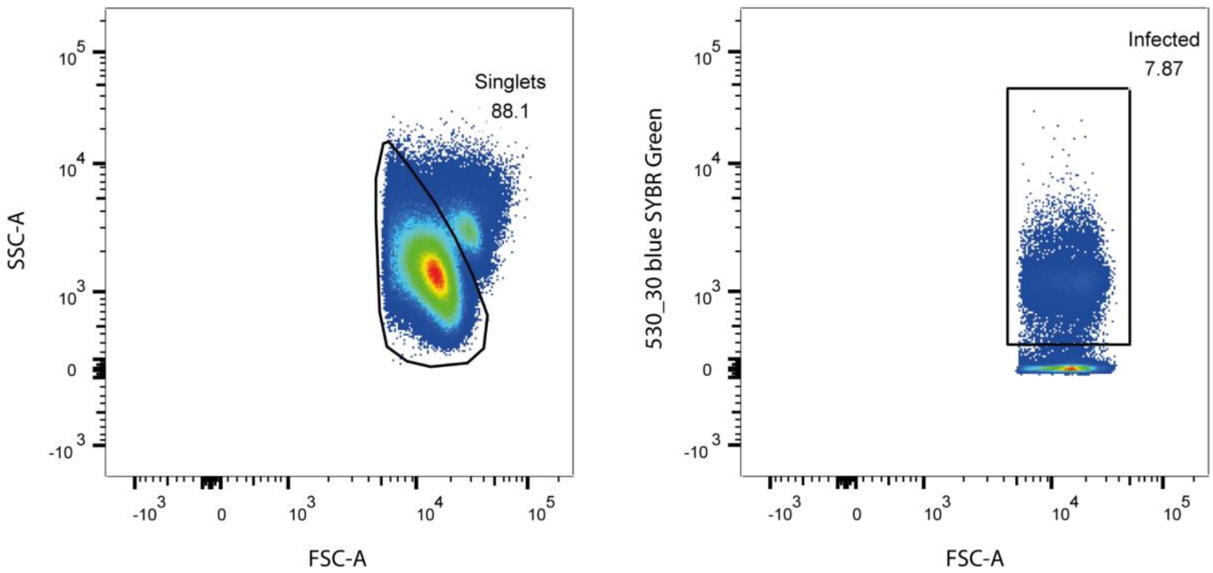

Supplementary Fig. 12. Flow cytometry gating strategy.
Samples were first gated for single cells by FSC-A and SSC-A. Infected cells were clearly labelled by SYBR Green.

1 Knuepfer, E., Napiorkowska, M., van Ooij, C. & Holder, A. A. Generating conditional gene knockouts in Plasmodium - a toolkit to produce stable DiCre recombinase-expressing parasite lines using CRISPR/Cas9. *Sci Rep* **7**, 3881, doi:10.1038/s41598-017-03984-3 (2017).

Source data for Supplementary Figure 1

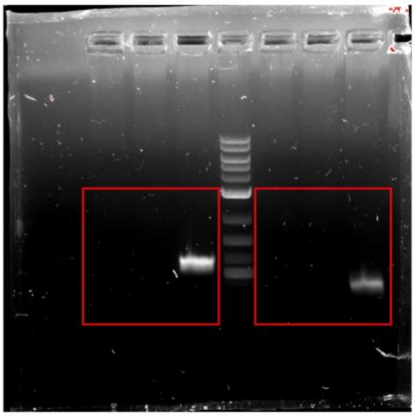

FIKK4.1 TurboID FIKK4.2 TurboID

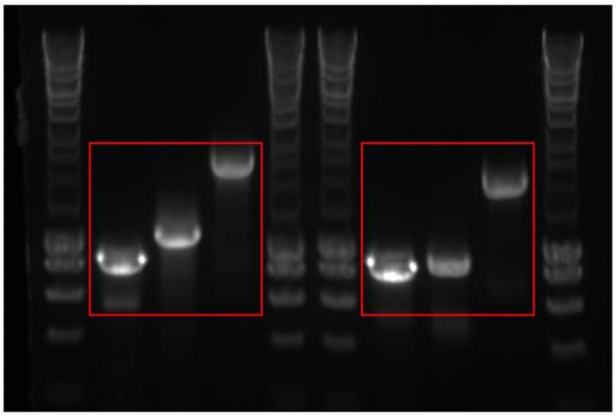

FIKK4.1 TurboID NF54 FIKK4.2 TurboID

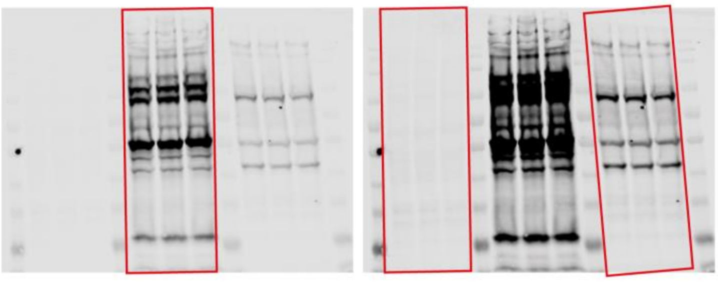

Streptavidin

NF54 FIKK4.1 TurboID FIKK4.2 TurboID

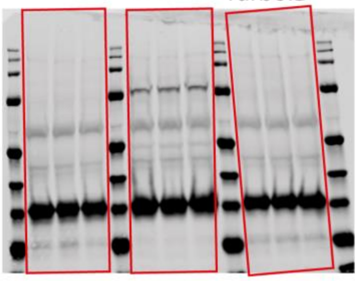

Anti-MAHRP1
Anti-V5

Source data for Supplementary Figure 2

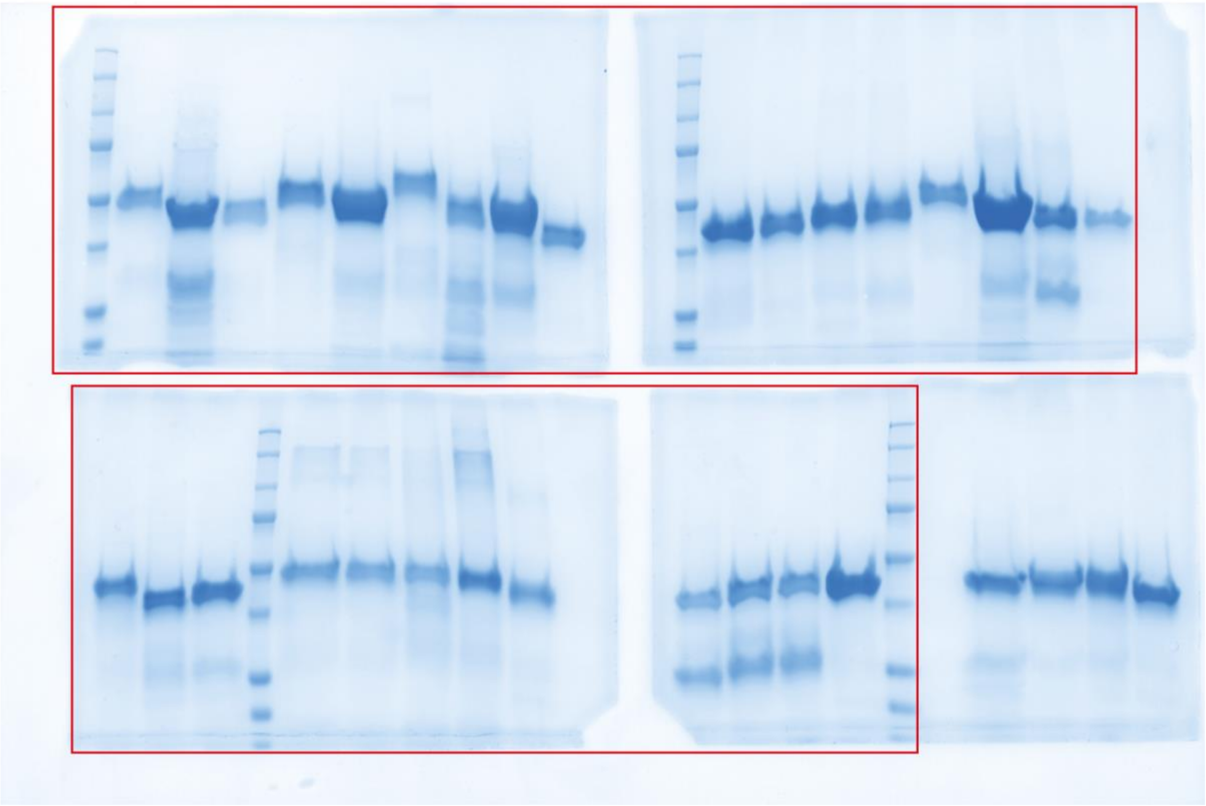

Source data for Supplementary Figure 11

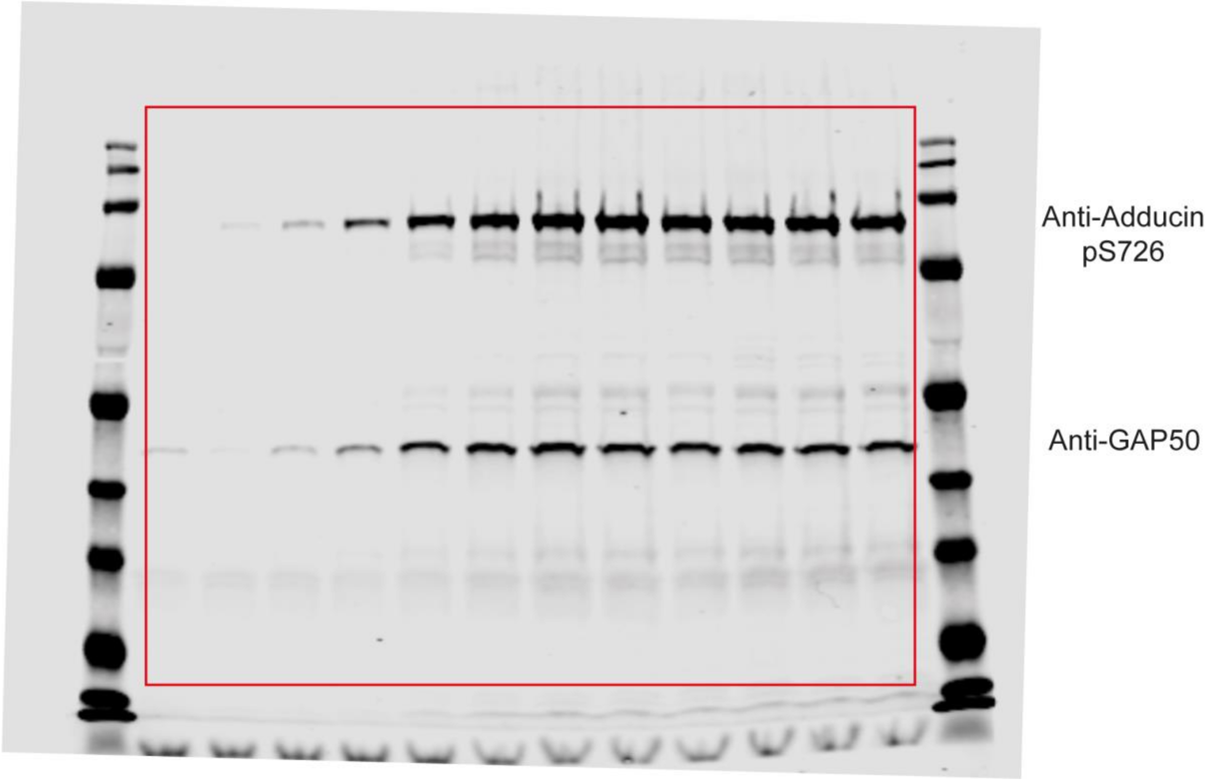

Supplement: Supplementary file 1 — Supplementary Figs. 1–12. [file 41564_2025_2017_MOESM1_ESM.pdf]
