# Supplementary material for: The fast-evolving FIKK kinase family of Plasmodium falciparum can be inhibited by a single compound
Source: Nat Microbiol. 2025 May 19;10(6):1463–83. doi: 10.1038/s41564-025-02017-4 (PMC12137140; doi:10.1038/s41564-025-02017-4)
Supplement: Supplementary file 2 — Reporting Summary [file 41564_2025_2017_MOESM2_ESM.pdf]

nature portfolio

Corresponding author(s): Moritz Treeck

Last updated by author(s): Mar 18, 2025

Reporting Summary

Nature Portfolio wishes to improve the reproducibility of the work that we publish. This form provides structure for consistency and transparency in reporting. For further information on Nature Portfolio policies, see our Editorial Policies and the Editorial Policy Checklist.

Statistics

For all statistical analyses, confirm that the following items are present in the figure legend, table legend, main text, or Methods section.

n/a	Confirmed	
☐	☒	The exact sample size (*n*) for each experimental group/condition, given as a discrete number and unit of measurement
☐	☒	A statement on whether measurements were taken from distinct samples or whether the same sample was measured repeatedly
☐	☒	The statistical test(s) used AND whether they are one- or two-sided<br>*Only common tests should be described solely by name; describe more complex techniques in the Methods section.*
☒	☐	A description of all covariates tested
☐	☒	A description of any assumptions or corrections, such as tests of normality and adjustment for multiple comparisons
☐	☒	A full description of the statistical parameters including central tendency (e.g. means) or other basic estimates (e.g. regression coefficient) AND variation (e.g. standard deviation) or associated estimates of uncertainty (e.g. confidence intervals)
☐	☒	For null hypothesis testing, the test statistic (e.g. *F*, *t*, *r*) with confidence intervals, effect sizes, degrees of freedom and *P* value noted<br>*Give P values as exact values whenever suitable.*
☒	☐	For Bayesian analysis, information on the choice of priors and Markov chain Monte Carlo settings
☒	☐	For hierarchical and complex designs, identification of the appropriate level for tests and full reporting of outcomes
☐	☒	Estimates of effect sizes (e.g. Cohen's *d*, Pearson's *r*), indicating how they were calculated

Our web collection on statistics for biologists contains articles on many of the points above.

Software and code

Policy information about availability of computer code

Data collection	FACSDiva v8.0.1, Nikon Elements v4.30.02, Xcalibur v4.2.28.14
Data analysis	Cytoscape v3.10.1, GraphPad Prism V10, FlowJo v10.8.2, MaxQuant v2.0.3.1, Perseus v1.5.0.9, Jalview 2.11.3.2, FIJI v2.1.0/1.53c, PyMOL 2.5.4, ImageLab 6.1, Haddock 2.4, IQ-TREE2 v2.0.7, ComplexHeatmap v2.12.1, ggtree v3.5.1.902, ggseqlogo v0.1, ggmsa v1.2.3, bio3d v2.4-4, bcftools v1.15.0, trimAI v2.0.<br>CCP4 version 8, XIA2 3.17.0, DIALS 3.17.0, PHASER 2.8.3, REFMAC 5.8.0430, COOT 0.9.8<br>SPR data were analysed using the Biacore Insight Evaluation Software.

For manuscripts utilizing custom algorithms or software that are central to the research but not yet described in published literature, software must be made available to editors and reviewers. We strongly encourage code deposition in a community repository (e.g. GitHub). See the Nature Portfolio guidelines for submitting code & software for further information.

Data

Policy information about availability of data

All manuscripts must include a data availability statement. This statement should provide the following information, where applicable:

- Accession codes, unique identifiers, or web links for publicly available datasets
- A description of any restrictions on data availability
- For clinical datasets or third party data, please ensure that the statement adheres to our policy

The mass spectrometry proteomics data have been deposited to the ProteomeXchange Consortium via the PRIDE113 partner repository with the dataset identifier PXD048966. The crystal structure of PfFIKK13149-561_D379N with Nb2G9, Nb9F10 and ATPγS is available from the Protein Data Bank under the accession code 9EMY. Gene sequences and annotations for P. falciparum 3D7 were acquired from PlasmoDB.org (v46)13 and human sequences were acquired from Uniprot.org (2023)86. RNA sequencing data from Hoeijmakers et al. (accession number GSE66185) available on PlasmoDB (www.PlasmodDB.org) was also used. The Pf3K project dataset used to identify genetic variants in fikk genes is available at the following address (www.malariagen.net/projects/parasite.pf3k)29. Source data in the form of unprocessed gels and western blots corresponding to Figs. 1c, 6d, 6e, 6f and Supplementary Figs. 1a, 1b, 2, 11c are available with the article.

Research involving human participants, their data, or biological material

Policy information about studies with human participants or human data. See also policy information about sex, gender (identity/presentation), and sexual orientation and race, ethnicity and racism.

Reporting on sex and gender	N/A
Reporting on race, ethnicity, or other socially relevant groupings	N/A
Population characteristics	N/A
Recruitment	N/A
Ethics oversight	N/A

Note that full information on the approval of the study protocol must also be provided in the manuscript.

Field-specific reporting

Please select the one below that is the best fit for your research. If you are not sure, read the appropriate sections before making your selection.

☒ Life sciences ☐ Behavioural & social sciences ☐ Ecological, evolutionary & environmental sciences

For a reference copy of the document with all sections, see nature.com/documents/nr-reporting-summary-flat.pdf

Life sciences study design

All studies must disclose on these points even when the disclosure is negative.

Sample size	Sample sizes are described in figure legends and methods. No statistical method was used to predetermine sample size.<br>In most instances we performed experiments in biological triplicates, unless otherwise stated.<br>Only one instance (Extended Data Fig. 4d, comparing "no substrate" and "FIKK13_4_Y10A") yielded a borderline p-value of close to 0.05, with a non-significant p-value of 0.0760. However, the data clearly show that FIKK13 loses its ability to phosphorylate peptide 4 when the tyrosine is mutated to alanine, confirming its tyrosine specificity. In all other cases, the results were definitive, with no indication of being overpowered or underpowered experiments.<br>For flow cytometry, parasitemia was determined by measuring the number of parasite-infected cells in a total of 100,000 red blood cells.
Data exclusions	As stated in the material and methods, for the proximity-labelling experiments we removed non-biotinylated peptides and biotinylated peptides present only in the NF54 sample (wild type) from the analysis as they represent background peptides binding to the beads.<br>Other than that, no data were excluded.
Replication	The number of repeats for each experiments is given either in the figure legends or in the methods.<br>The proximity-labelling experiment has been performed in biological triplicate for each condition. Additionally, each replicate was cultured in a blood coming from different donors. The high consistence between replicates, and the agreement with our previously published dataset (PMID:32284562) provided high level of confidence to the sites which were observed to be significantly enriched in the TurboID-fusion samples.<br>Peptide libraries experiments were mostly performed once per kinase. Some kinases have been tested several times on the peptide libraries, each time giving consistent results. Additionally, the consistency of preferred phosphorylation motifs between FIKK kinases from two different species give a high degree of confidence in the data.<br>Binding affinity of cyclic peptides identified as binding to FIKK13 were measured in at least 2 independent replicates.<br>ADP-Glo assays measuring kinase activity in the presence of different substrates were always performed in biological triplicates, except for Fig.

	7a where each inhibitor was tested in 6 technical replicates.<br>Screening of the PKIS library against FIKK8 was performed in biological duplicates. Structural-Activity Relationship assay and IC50 determination of compounds were performed in biological triplicates.<br>ATP levels in the ATP-depletion optimisation steps were measured in biological triplicates.
Randomization	Randomisation was not relevant to this study as no subjective judgments were required about which data to include, exclude, or measure.
Blinding	Investigators were not blinded during data collection and/or analysis as measurements were performed on quantitative endpoints that are not subject to investigator bias.

Reporting for specific materials, systems and methods

We require information from authors about some types of materials, experimental systems and methods used in many studies. Here, indicate whether each material, system or method listed is relevant to your study. If you are not sure if a list item applies to your research, read the appropriate section before selecting a response.

Materials & experimental systems

n/a	Involved in the study	
☐	☒	Antibodies
☐	☒	Eukaryotic cell lines
☒	☐	Palaeontology and archaeology
☐	☒	Animals and other organisms
☒	☐	Clinical data
☒	☐	Dual use research of concern
☒	☐	Plants

Methods

n/a	Involved in the study	
☒	☐	ChIP-seq
☐	☒	Flow cytometry
☒	☐	MRI-based neuroimaging

Antibodies

Antibodies used	Anti-HA high affinity antibodies (Clone 3F10, Roche, Lot number: 62572200, Cat. no: 11867431001). Dilution WB: 1 in 1,000, IFA: 1 in 1,000.<br>Anti-V5 (SV5-Pk1, Abcam, Cat. no: ab27671, Lot no: GR3337308-16). Dilution WB: 1 in 1,000<br>Anti-MAHRP1 (not commercially available, gift from Julian Rayner and Lindsay Parish). Dilution WB: 1 in 2,000.<br>Anti-GAP50 (not commercially available, gift from Julian Rayner). Dilution WB: 1 in 2,000.<br>Anti Adducin phospho-726 (Abcam, Cat. no ab53093, Lot no: GR81840-3). Dilution WB: 1 in 1,500, IFA: 1 in 1,000.<br>Anti-SBP1 (not commercially available, gift from Tobias Spielmann). Dilution IFA: 1 in 10,000.<br>Anti-GAPDH MAb 7.2 (European Malaria Reagent Repository (EMRR: www.malariaresearch.eu)). Dilution WB: 1 in 10,000.<br>Anti-FIKK4.2 MAb 126 (European Malaria Reagent Repository (EMRR: www.malariaresearch.eu)). Dilution IFA: 1 in 1,000.<br>Anti-Biotin Polyclonal Ab (Bethyl Laboratories, Cat. no: 150-109A)<br>Anti-Biotin Polyclonal Ab (Abcam, Cat. no: ab53494)<br>Anti-c-Myc MAb (Clone 9E10, ThermoFisher Scientific, Cat. no: MA1-980). Dilution ELISA: 1 in 2,000<br>Anti-FIKK13 nanobodies, described in this paper
Validation	Commercially available antibodies were validated by the suppliers. All commercially available antibody had validation statement available on the website of the suppliers.<br>Anti-HA high affinity antibodies (Clone 3F10, Roche, Lot number: 62572200, Cat. no: 11867431001) https://www.sigmaaldrich.com/GB/en/product/roche/roahaha?srsltid=AfmBOopff2YHT2mLNVsXDGFweR0ETGiame4ZWRznhmDod33MXgeDmqej<br>Anti-V5 (SV5-Pk1, Abcam, Cat. no: ab27671, Lot no: GR3337308-16) https://www.abcam.com/en-us/products/primary-antibodies/v5-tag-antibody-sv5-pk1-ab27671?srsltid=AfmBOor-BHxHZhU2Cq1LQr6hwJPl_4Q1w99na1YfilVUD52v8pmlllcT<br>Anti Adducin phospho-726 (Abcam, Cat. no ab53093, Lot no: GR81840-3) https://www.abcam.com/en-us/products/primary-antibodies/alpha-adducin-phospho-s726-antibody-ab53093?srsltid=AfmBOoo6HoOf5t5Cj4LkjSprZ_g52JEEkWEvt2SarZ9ulfGeoOHpUn_V<br>Anti-c-Myc MAb (Clone 9E10, ThermoFisher Scientific, Cat. no: MA1-980) https://www.thermofisher.com/antibody/product/c-Myc-Antibody-clone-9E10-Monoclonal/MA1-980<br>Anti-MAHRP1 - Antibody validation unpublished. In a previous publication we saw a single band in Plasmodium infected cells only (not uninfected RBCs) by western blot and IFA probed with the antibody showed staining of maurer's clefts, where MAHRP1 is known to localise, and no staining of uninfected RBC, confirming specificity. In this publication, we observe the same single band at the expected size by western blot.<br>Anti-GAP50 has been characterised previously by immunoblot and IFA (doi: 10.1016/j.molbiopara.2006.01.009).<br>Anti-SBP1 has been characterised previously by immunoblot (PMCID: PMC4864081).<br>Anti-GAPDH MAb 7.2 has been characterised previously by immunoblot and IFA (doi: 10.1016/0166-6851(83)90025-7) http://www.malariaresearch.eu/reagents/monoclonal-antibody/72-anti-gapdh.<br>Anti-FIKK4.2 MAb 126 has been characterised previously by immunoblot and IFA (doi: 10.1016/j.ijpara.2014.01.003) http://www.malariaresearch.eu/reagents/monoclonal-antibody/126-anti-fikk42.<br>Both anti-GAPDH and anti-FIKK4.2 monoclonal antibodies were provided by the European Malaria Reagent Repository.<br><br>Anti-FIKK13 nanobodies were selected by ELISA and binding was confirmed by the fact that the complex FIKK13+nanobodies eluted at a higher molecular weight than FIKK13 alone or FIKK13 + 1 nanobody. Moreover, both nanobodies can be observed bound to

FIKK13 in the crystal structure.

Eukaryotic cell lines

Policy information about cell lines and Sex and Gender in Research

Cell line source(s)	The NF54 DiCre line was made within our lab by inserting the DiCre locus into the transmissible lab-strain NF54 - ref -Tiburcio, M. et al. (2019). "A Novel Tool for the Generation of Conditional Knockouts To Study Gene Function across the Plasmodium falciparum Life Cycle." MBio 10(5). doi: 10.1128/mBio.01170-19<br><br>Plasmodium knowlesi parasites adapted for cell culture were obtained from Rob Moon - ref - Moon, R. W. et al. "Adaptation of the genetically tractable malaria pathogen Plasmodium knowlesi to continuous culture in human erythrocytes". Proc Natl Acad Sci U S A 110, 531-536, doi:10.1073/pnas.1216457110 (2013).<br><br>Commercial E. coli BL21-Gold (DE3) cells (Stratagene) were used for recombinant protein expression.
Authentication	None were authenticated
Mycoplasma contamination	All cell lines (Plasmodium and bacteria used for recombinant expression) were not tested for mycoplasma contamination.
Commonly misidentified lines (See ICLAC register)	No commonly misidentified lines were used.

Animals and other research organisms

Policy information about studies involving animals; ARRIVE guidelines recommended for reporting animal research, and Sex and Gender in Research

Laboratory animals	Llama Glama, 6 years old
Wild animals	No
Reporting on sex	A single animal was immunised to generate nanobodies. The sex of the animal is not indicated.
Field-collected samples	No
Ethics oversight	An established protocol with animal handling carried out by trained personnel under the Home Office Project Licence PA1FB163A was followed.

Note that full information on the approval of the study protocol must also be provided in the manuscript.

Plants

Seed stocks	N/A
Novel plant genotypes	N/A
Authentication	N/A

Flow Cytometry

Plots

Confirm that:

☒ The axis labels state the marker and fluorochrome used (e.g. CD4-FITC).

☒ The axis scales are clearly visible. Include numbers along axes only for bottom left plot of group (a 'group' is an analysis of identical markers).

☒ All plots are contour plots with outliers or pseudocolor plots.

☒ A numerical value for number of cells or percentage (with statistics) is provided.

Methodology

Sample preparation	All Flow Cytometry samples in this study were Plasmodium infected red blood cells. The blood was obtained from the National Health Service Blood and Transplant (NHSBT) service.<br><br>For the half-maximal effective concentration (EC50) of FIKK inhibitors, samples were fixed in 2% paraformaldehyde (PFA) + 0.2% glutaraldehyde (GA) in PBS for 1 hour in the dark at 4 degrees. Fixative was subsequently washed out with PBS and samples were stained with SYBR Green for 30 minutes in the dark at 37 degrees.
Instrument	BD LSRFortessa flow cytometer (Becton Dickinson)
Software	Collected with FACSDiva, analysed with FlowJo10
Cell population abundance	Cells were not sorted, just analysed.<br>For parasitemia measurement for EC50 assessment, one hundred thousand singlet events for each sample were measured.<br>For parasitemia measurement for ATP-delpetion optimisation, three hundred thousand singlet events for each sample were measured.
Gating strategy	Samples were first gated for single cells by FSC-A and SSC-A.<br>Infected cells were clearly labelled by SYBR Green

☒ Tick this box to confirm that a figure exemplifying the gating strategy is provided in the Supplementary Information.
