# Supplementary material for: The fast-evolving FIKK kinase family of Plasmodium falciparum can be inhibited by a single compound
Source: Nat Microbiol. 2025 May 19;10(6):1463–83. doi: 10.1038/s41564-025-02017-4 (PMC12137140; doi:10.1038/s41564-025-02017-4)
Supplement: Supplementary file 5 — Data collection and refinement statistics (molecular replacement) for PfFIKK13149-561_D379N+Nb2G9+Nb9F10+ ATPgS. [file 41564_2025_2017_MOESM5_ESM.pdf]

Supplementary Table 17. Data collection and refinement statistics (molecular replacement) for *Pf*FIKK13$^{149-561_D379N}$ + Nb2G9 + Nb9F10+ ATPγS.

	*Pf*FIKK13$^{149-561_D379N}$ + Nb2G9 + Nb9F10+ ATPγS
**Data collection**	
Space group	P 2_1
Cell dimensions	
a, *b*, *c* (Å)	82.40, 121.66, 151.06
α, β, γ (°)	90, 90.02, 90
Resolution (Å)	64.17–2.81(2.86-2.81)
R_{merge}	0.196(2.123)
R_{pim}	0.079(0.844)
$I / \sigma I$	6.8 (0.4)
Completeness (%)	99.7% (96.1%)
Multiplicity	7.03 (7.28)
$CC_{1/2}$	0.995 (0.615)
**Refinement**	
Resolution (Å)	2.81
No. reflections	72237
R_{work} / R_{free}	24.5% / 29.2%
No. atoms	19165
Protein	18999
Ligand/ion	124 AGS / 24 PE4
Water	18
B-factors	
Protein	73.1
Ligand/ion	75.3 AGS / 132 PE4
Water	55.3
R.m.s. deviations	
Bond lengths (Å)	0.0061
Bond angles (°)	1.4940

Values in parentheses are for highest-resolution shell.
