# Supplementary figures and images for: The fast-evolving FIKK kinase family of Plasmodium falciparum can be inhibited by a single compound
Source: Nat Microbiol. 2025 May 19;10(6):1463–83. doi: 10.1038/s41564-025-02017-4 (PMC12137140; doi:10.1038/s41564-025-02017-4)

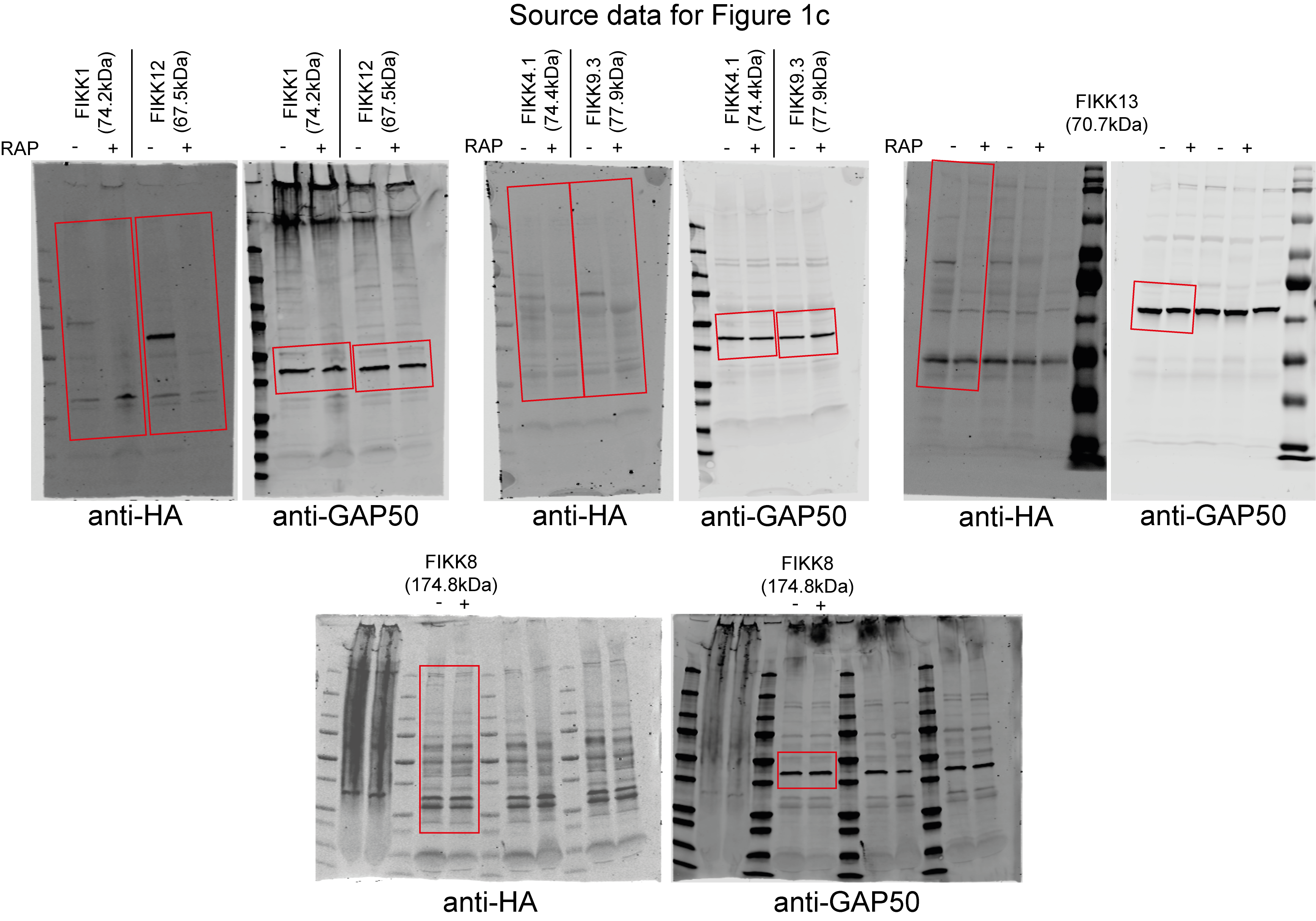

Supplement: Supplementary file 6 — Unprocessed western blots for Fig. 1c. [file 41564_2025_2017_MOESM6_ESM.tif]

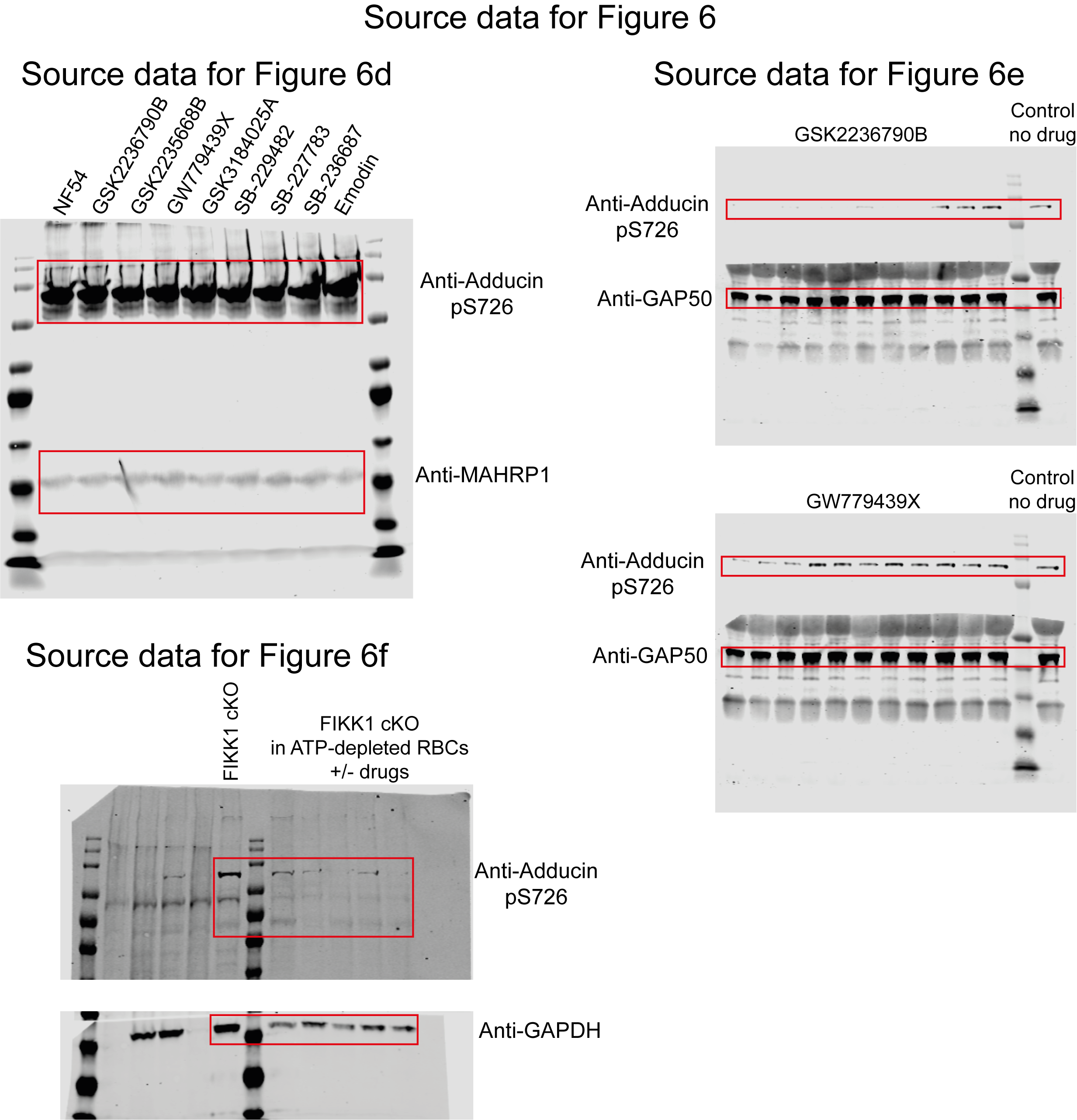

Supplement: Supplementary file 7 — Unprocessed western blots for Fig. 6d–f. [file 41564_2025_2017_MOESM7_ESM.tif]
